# Evaluation of Lignan Compound Content and Bioactivity of Raw Omija and Sugared Omija in Serum of Sprague Dawley Rat

**DOI:** 10.3390/foods8090373

**Published:** 2019-09-01

**Authors:** Sung-Hyun Hwa, Su-Jung Yeon, Go-Eun Hong, Won-Young Cho, Ha-Jung Lee, Ji-Han Kim, Chi-Ho Lee

**Affiliations:** 1 Department of Food Science and Biotechnology of Animal Resources, Konkuk University, Seoul 05029, Korea; 2 Department of Agricultural, Life and Environmental Science, Tottori University, Tottori 680-8550, Japan; 3 Lab supervisor, Factory, Royal Canin Korea, Jeollabuk-do 54325, Korea; 4 AgResearch (Grasslands Research Centre), Palmerston North 4442, New Zealand

**Keywords:** omija, *Schizandra chinensis*, sugared omija, byproduct, renin

## Abstract

This study evaluated the lignan contents of raw omija (R) and sugared omija (S), byproducts discarded after the use of raw omija, by HPLC and determined their bioactivity by feeding rats R and S for eight weeks. S retained 63% more lignan than R. Body weight gains in the raw omija-fed group (RO) and sugared omija-fed group (SO) decreased significantly compared to that of the control group (CON, *p* < 0.05). Glucose and aspartate aminotransferase levels in the serum of the experimental groups were lower than those in CON, especially in SO (*p* < 0.05). The amount of atrial natriuretic peptide in RO decreased significantly compared to that in CON (*p* < 0.05). The renin activity in RO increased and that in SO decreased compared to the same in CON (*p* > 0.05). Therefore, it was suggested that sugared omija contains lignan compounds and potentially contributes to bioactivity in that it decreases blood glucose levels and blood pressure.

## 1. Introduction

Omija (*Schizandra chinensis*) is a Korean fruit that offers five tastes, i.e., it is sweet, salty, bitter, sour, and hot [1]. These tastes are due to several compounds such as organic acids, glucose, and fructose [2]. The fruit also includes anthocyanin in the peel, resulting in its red color [2]. It has been traditionally used as a natural medicine to treat fatigue, as well as for its antipyretic action, and to improve visual activity, in addition to being used as a food additive for beverages, fruit punch, and sugared omija [3].

The functional applications of omija originate from constituent lignan compounds such as schizandrins and gomisins [4]. Accordingly, omija exhibits antioxidant activities [5], inhibits nephrotoxicity [6], and offers liver protection [7]. Lee et al. [4] reported that the main lignan of omija is schizandrin and the highest content of schizandrin is in omija seeds. However, the seed and peel of omija are discarded after it is used for preparing sugared omija or after the juice is extracted [4], and these byproducts also pose environmental problems [8].

Many researchers have attempted to utilize such byproducts, such as when Makgeolli byproducts were used as cosmetic materials [9] and flavonoid extract from onion peels as functional food material [10]. As for omija, Lee et al. [4] determined the chemical components in different parts of omija and the quality characteristics of omija seed oils by roasting and extraction. Various studies on the fruit itself have been reported consistently, but little is known about the seed and peel of omija.

Recently, the effects of several bioactive compounds have been evaluated as index substances. Serum hepatic enzymes such as aspartate aminotransferase (AST) have been used in the diagnosis and monitoring of liver disease [11]. Atrial natriuretic peptide (ANP) has been reported to decrease arterial pressure [12]. Renin converts angiotensinogen to angiotensin, which is associated with vasoconstriction [13,14]. Meanwhile, Jo et al. [15] researched α-amylase and α-glycosidase inhibitions to clarify the anti-hyperglycemic effect of omija fruit.

In this study, sugared omija produced as the byproduct of juice extraction processing and raw omija fruit were evaluated for their lignan compound contents using high-performance liquid chromatography (HPLC). Furthermore, the AST, ANP, and renin activities were compared by serum analysis for Sprague Dawley (SD) rats that were fed sugared omija and raw omija fruit to investigate the bioavailability of discarded sugared omija.

## 2. Materials and Methods

### 2.1. Preparation of Samples

Raw omija and sugared omija were purchased in dry form from mungyoungmall (Mungyeong-si, Gyeongsangbuk-do, Korea) and ground using a blender (NFM-9960, NUC. CO. LTD., Daegu, Korea). Powdered samples used for the experiment were denoted as raw omija (R) and sugared omija (S). R and S contained 6.0% (*w/w*) and 5.0% (*w/w*) of moisture, 12.8% (*w/w*) and 7.0% (*w/w*) of crude protein, 18.2% (*w/w*) and 9.6% (*w/w*) of crude fat, and 2.5% (*w/w*) and 1.0% (*w/w*) of ash, respectively. 

### 2.2. Analysis of Lignan Compounds

Lignan compounds in omija samples were analyzed by HPLC (Agilent 1100 series, Agilent, Santa Clara, CA, USA) according to the method reported by Koo et al. [16] with slight modification. For analysis, the sample was diluted with MeOH and extraction was undertaken after shaking for 3 h at 40 °C. Then, the extract was centrifuged and the supernatant was collected. This process was repeated once more. After filtering with a 0.45-μm PVDF membrane syringe filter (PALL Life Sciences), the filtrate was subjected to HPLC analysis. Gradient elution was used with acetonitrile:water = 20%:80% (t = 0) to 100%:0% (t = 25) and the post time was 5 min. The flow rate was 1.0 mL/min and a C18 column (4.6 × 150 mm, 5 μm, Shiseido Fine Chemicals, Tokyo, Japan) was used at 25 °C. Absorbance was detected at 254 nm.

### 2.3. Animal Experiments

All animal experiments were approved by the Institutional Animal Care and Use Committee (IACUC approval no. KU17133). Five-week-old male SD rats were obtained from Saeronbio Inc. (Uiwang-si, Gyeonggi-do, Korea) and six rats were assigned to each of the groups, the control group (CON), raw omija-fed group (RO), and sugared omija-fed group (SO), which denoted the diet of the rats (Feedlab, Gyeonggi-do, Korea), as summarized in Table 1. The diet was fed to the rats at a rate of 20 g/rat/day and tap water was available ad libitum. The losses of offered solid pellet diet were weighed in the next morning before offering a new 20 g pellet diet. All rats were allowed an acclimation period of 1 week and the experimental diet feeding period was 8 weeks at 22 ± 1 °C, 50 ± 5% humidity, and a 12 h light/dark cycle. All rats fasted for 24 h prior to sacrifice. Blood was collected from the abdominal vein and the organs were weighed.

### 2.4. Serum Analysis

Serum was obtained by centrifugation at 3000 rpm for 20 min at 4 °C after letting collected blood stand at 25 °C for 3 h prior to centrifugation, and it was analyzed by Green Cross Corp. (Korea).

### 2.5. Statistical Analysis

Results are expressed as mean ± standard deviation. All data were analyzed using the program SPSS for Windows (version 18.0, SPSS Inc., Chicago, IL, USA). The amount of lignan compounds was analyzed by t-tests, and animal experimental data were analyzed by the one-way analysis of variance (ANOVA). Significant differences (*p* < 0.05) were determined using Duncan’s multiple range tests.

## 3. Results

### 3.1. Composition of Lignan Compounds

**Figure 1:**
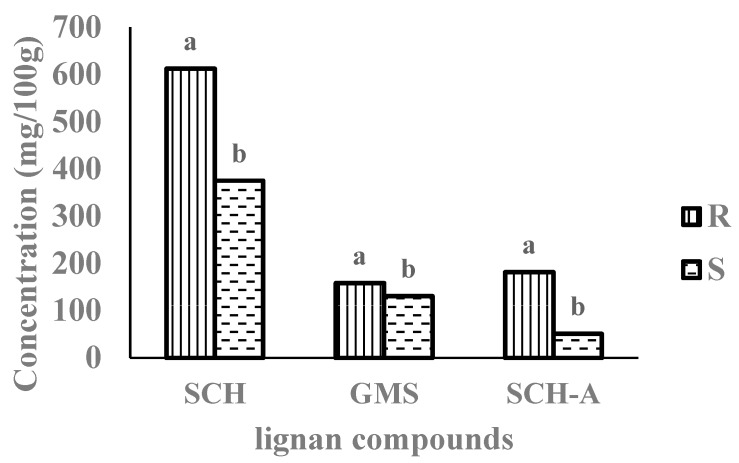
Amounts of lignan compounds in dried raw omija and sugared omija. All values denote mean. SCH—Schizandrin; GMS—Gomisin; SCH-A—Schizandrin A; R—0.5% raw omija powder-fed group; S—0.5% sugared omija powder-fed group. ^a,b^ superscripts with different letters are significantly different (*p* < 0.05).

Three kinds of lignan compounds were analyzed quantitatively in powders of raw omija and sugared omija (Figure 1). Raw omija and sugared omija contained significantly different amounts of schizandrin at about 600 mg/100 g and 400 mg/100 g, respectively (*p* < 0.05), but the gomisin contents did not show a significant difference (*p* > 0.05). A significant decrease in the schizandrin A amount was also noted for sugared omija compared to that in raw omija (almost 200 and 50 mg/100 g, respectively, *p* < 0.05). Altogether, the total amount of lignan compounds in sugared omija, the generally discarded byproduct, was 63% more than that in raw omija.

### 3.2. Animal Experiments

#### 3.2.1. Final Body Weight, Body Weight Gain, and Total Feed Intake

The feed intake was not significantly different among the groups during the experimental period (*p* > 0.05, Table 2). Body weight gains for the two experimental groups were significantly lower than that for the CON group (*p* < 0.05), and it affected the final body weight, but the latter remained within the normal range for body weight [17].

#### 3.2.2. Weight of Organs

There were no significant changes in the weights of the kidneys and spleens in any group (*p* > 0.05, Table 3). The weights of epididymal fat (EAT) did not show any significant difference, either, even though that of SO decreased by 6.7% more than that of CON (*p* > 0.05). However, the retroperitoneal fat (PAT) of the sugared omija-fed group weighed the lowest among all groups and decreased by 31% more than that for the raw omija-fed group (*p* < 0.05). 

#### 3.2.3. Serum Analysis

The level of serum glucose in SO and RO was significantly lower (*p* < 0.05 and *p* > 0.05, respectively) than that in CON (Table 4). The amounts of aspartate aminotransferase (AST) and atrial natriuretic peptide (ANP) in RO and SO were lower than that in CON, but the AST level in RO and ANP in SO did not show a significant decrease compared to that in CON (*p* > 0.05). The level of rennin in RO increased and that in SO decreased compared to that in CON, but the values did not show a significant difference (*p* > 0.05). However, the renin activity of SO showed a significant decrease compared to that of RO (*p* < 0.05).

## 4. Discussion

Schizandrins, a group of predominant lignan compounds, primarily exist in seeds of the omija fruit [4]. 

As seen in Figure 1, the amounts of lignan compounds in S were more than half of those in R. The seed and peel of omija remained in the sugared omija, and, thus, the lignan compound content recorded for sugared omija was high, suggesting that the lignan compounds were primarily released from the seed and peel. After identifying a high amount of lignan compounds in the discarded S, the animal experiment was proceeded to investigate the bioactivity of the compounds in R and S.

Han et al. [18] reported a decrease in the body weight gain in 3% omija water extract-fed SD rats compared to that in deionized water-fed SD rats. In their study, they used 4-week-old male SD rats and raised them for only 4 weeks. In this study, 5-week-old SD rats were used and the experimental period was 8 weeks; therefore, significant differences are justifiable. 

According to Jo et al. [15], water extract of omija pulp/skin (OPE) statistically and significantly reduced blood glucose in vivo (*p* < 0.05), and omija seed water extract (OSE) decreased it compared to their control. They proposed that this phenomenon is related to the inhibition activity of α-glucosidase, the enzyme that converts starch to maltose in the small intestine, wherein OPE exhibits a higher inhibition activity than OSE (*p* < 0.05). Furthermore, Cho et al. [19] reported high α-amylase and α-glucosidase inhibition activities in omija extract using water and 60% ethanol. Because of these characteristics of omija, omija-fed groups might show decreased blood glucose than that of CON.

When the liver is damaged, AST is released to the blood. Omija-fed groups maintained a lower serum AST than CON, and the sugared omija-fed group showed a significantly lower AST amount, suggesting that the liver damage can potentially be treated by the intake of sugared omija. Indeed, there have been several in vivo reports about the hepatoprotective effect of omija in liver-damaged animals [20,21]. However, this report is the first recording lower AST levels in rats with no induced liver damage. Song et al. [20] reported anthocyanin and lignans in omija ethanol extract, and suggested that this is related to an improved liver in high-fat-diet-induced fatty liver rat. In this study, RO and SO also contained lignan compounds, which could be expected to induce liver repair.

ANP releases Na+ through urine [22] and is involved in vasodilation. Follenius et al. [23] mentioned that increased ANP parallels decrease renin activity in plasma owing to inhibited renal renin release. Renin is related to vasoconstriction because of angiotensin, which is converted from angiotensinogen by renin, so the regulation of renin activity is important to hypertension patients. In terms of renin activity, the SO group was lower than the CON group and decreased significantly more than that of RO (*p* < 0.05), and it is potentially more appropriate as a treatment for blood pressure than raw omija. In ANP, SO increased more than RO and is not significantly different from both RO or CON. In the context of ANP amount and renin activity, sugared omija might act as a potential treatment measure for hypertension patients and may be better than raw omija.

There are several bioactive compounds, such as anthocyanin and organic acid, as well as lignans, in omija. Glycoside, such as the complex of polyphenol glycoside (for example, anthocyanin), in omija might be decomposed and aglycon would be revealed by its enzyme for 100 days of the sugaring period. Therefore, the bioavailability of bioactive compounds would be increased, and it might be related to equivalent or more potent biological activity of sugared-omija than the raw one.

Meanwhile, bioactivities of lignan on hepatoprotective activity, anti-inflammatory activity, antioxidative activities, anticancer activity, and cardiovascular diseases have been reported [24]. Jo et al. [15] reported in vitro and in vivo anti-hyperglycemic effects of omija fruit, but the effect of lignan compounds on diabetes may not have been reported.

In conclusion, sugared omija can be used as a functional ingredient (e.g., desserts, patient meal) for hyperglycemic, hypertension, and/or liver disease patients or people who want to eat a healthier diet because of its bioactivity on decreasing the serum glucose level and degree of liver damage. Moreover, it was also in the index of hypertension. However, in order to establish an accurate amount for human interventions, additional research should be carried out in the efficacy and innocuousness of the sugared omija product, to establish the recommended and tolerable dosages.

## Figures and Tables

**Table 1:** Composition of diet.

Ingredient (g)	CON	RO	SO
Casein	200.00	199.32	199.63
Sucrose	100.00	100.00	100.00
Dextrose	132.00	132.00	132.00
Corn Starch	397.49	394.14	393.39
Cellulose	50.00	50.00	50.00
Soybean Oil	70.00	69.03	69.49
Raw omija powder		5.00	
Sugared omija powder			5.00
TBHQ	0.01	0.01	0.01
Mineral	35.00	35.00	35.00
Vitamin mix	10.00	10.00	10.00
L-Cystine	3.00	3.00	3.00
Choline Bitartrate	2.50	2.50	2.50
Total (g)	1000.00	1000.00	1000.00
Kcal/kg	4000.00	4000.00	4000.00

CON—control diet-fed group; RO—0.5% raw omija powder-fed group; SO—0.5% sugared omija powder-fed group.

**Table 2:** Body weight and feed intake.

	CON	RO	SO
Final body weight (g)	400.00 ± 10.56 ^a^	385.33 ± 7.63 ^b^	384.00 ± 4.47 ^b^
Body weight gain (g)	234.17 ± 10.76 ^a^	217.67 ± 9.09 ^b^	217.33 ± 5.72 ^b^
Feed intake (g)	784.67 ± 4.08 ^NS^	781.65 ± 2.49	783.00 ± 0.00

All values denote mean ± SD. CON—control diet-fed group; RO—0.5% raw omija powder-fed group; SO—0.5% sugared omija powder-fed group. ^a,b^ superscripts with different letters are significantly different (*p* < 0.05). ^NS^ not significant.

**Table 3:** Organ weights.

	CON	RO	SO
kidney (g)	2.56 ± 0.19 ^NS^	2.67 ± 0.37	2.79 ± 0.08
spleen (g)	0.81 ± 0.10 ^NS^	0.78 ± 0.08	0.82 ± 0.10
epididymal fat (g)	5.67 ± 1.50 ^NS^	5.58 ± 2.42	5.29 ± 1.54
retroperitoneal fat (g)	8.12 ± 1.25 ^ab^	9.08 ± 2.61 ^a^	6.20 ± 0.92 ^b^

All values denote mean ± SD. CON—control diet-fed group; RO—0.5% raw omija powder-fed group; SO—0.5% sugared omija powder-fed group. ^a,b^ superscripts with different letters are significantly different (*p* < 0.05). ^NS^ not significant.

**Table 4:** Serum analysis.

	CON	RO	SO
Glucose (mg/dL)	144.17 ± 38.32 ^a^	127.67 ± 24.34 ^ab^	104.83 ± 20.18 ^b^
AST (U/L)	185.50 ± 66.70 ^a^	148.33 ± 25.30 ^ab^	125.50 ± 18.11 ^b^
ANP (pg/mL)	75.35 ± 25.59 ^a^	42.18 ± 21.91 ^b^	67.01 ± 18.88 ^ab^
Renin activity (ng/mL/h)	34.18 ± 13.30 ^ab^	40.12 ± 11.14 ^a^	24.14 ± 8.49 ^b^

All values denote mean ± SD. CON—control diet-fed group; RO—0.5% raw omija powder-fed group; SO—0.5% sugared omija powder-fed group; AST—aspartate aminotransferase; ANP—atrial natriuretic peptide. ^a,b^ superscripts with different letters are significantly different (*p* < 0.05).
